# Influence of Addition of Antibiotics on Chemical and Surface Properties of Sol-Gel Coatings

**DOI:** 10.3390/ma15144752

**Published:** 2022-07-07

**Authors:** Beatriz Toirac, Amaya Garcia-Casas, Miguel A. Monclús, John J. Aguilera-Correa, Jaime Esteban, Antonia Jiménez-Morales

**Affiliations:** 1 Materials Science and Engineering and Chemical Engineering Department, Carlos III University of Madrid, 28911 Madrid, Spain; amayagarciacasas@gmail.com (A.G.-C.); toni@ing.uc3m.es (A.J.-M.); 2 CIDETEC, Basque Research and Technology Alliance (BRTA), 20014 Donostia-San Sebastián, Spain; 3 Micro- and Nano-Mechanics Department, Madrid Institutes for Advanced Studies (IMDEA)—Materials, 28906 Madrid, Spain; miguel.monclus@imdea.org; 4 Clinical Microbiology Department, IIS-Fundación Jiménez Díaz, UAM, 28040 Madrid, Spain; john.aguilera@fjd.es (J.J.A.-C.); jesteban@fjd.es (J.E.); 5 CIBERINFEC, ISCIII—CIBER de Enfermedades Infecciosas, Instituto Carlos III, 28029 Madrid, Spain; 6 Alvaro Alonso Barba Technological Institute of Chemistry and Materials, Carlos III University of Madrid, 28911 Madrid, Spain

**Keywords:** antibiotics-loaded sol-gel coatings, AFM, SEM, solid-state ^29^Si-NMR spectroscopy, Fourier-transform infrared spectroscopy

## Abstract

Infection is one of the most common causes that leads to joint prosthesis failure. In the present work, biodegradable sol-gel coatings were investigated as a promising controlled release of antibiotics for the local prevention of infection in joint prostheses. Accordingly, a sol-gel formulation was designed to be tested as a carrier for 8 different individually loaded antimicrobials. Sols were prepared from a mixture of MAPTMS and TMOS silanes, tris(tri-methylsilyl)phosphite, and the corresponding antimicrobial. In order to study the cross-linking and surface of the coatings, a battery of examinations (Fourier-transform infrared spectroscopy, solid-state ^29^Si-NMR spectroscopy, thermogravimetric analysis, SEM, EDS, AFM, and water contact angle, thickness, and roughness measurements) were conducted on the formulations loaded with Cefoxitin and Linezolid. A formulation loaded with both antibiotics was also explored. Results showed that the coatings had a microscale roughness attributed to the accumulation of antibiotics and organophosphites in the surface protrusions and that the existence of chemical bonds between antibiotics and the siloxane network was not evidenced.

## 1. Introduction

Orthopedic implant-associated infections (IAI) are especially challenging for orthopedic trauma services [1]. Infections are caused by bacteria or fungi attached to the implant surface, leading to biofilm formation on the implant surface. Orthopedic IAI can have devastating consequences for patients and represents a significant economic cost in hospital expenses. When IAI occurs, the traditional protocol can include surgery (irrigation and debridement, obliteration of dead space, intravenous administration of antibiotics, and biomaterial removal) associated with a prolonged systemic antibiotic treatment [2]. The recovery chances of limb functionality, even if the infected implant is successfully removed, are quite limited. Sometimes, these procedures usually lead to a bad outcome, such as arthrodesis, amputation, suppressive treatment, and even death [3,4,5]. Therefore, avoiding the growth of nosocomial pathogens can be more effective than trying to eliminate the biofilm [6].

The orthopedic IAI prevention by systemic administration of antibiotics probably has reached its limit in its effectiveness, and the increasing number of resistant organisms could be a problem in the future. To overcome this problem, possibly will be necessary to use a broader spectrum antibiotics with, in some cases, an increased number of side effects [6]. In addition, after prosthetic implantation, the tissue may be damaged, avascular, or even necrotic. These consequences locally decrease the antibiotic concentration systemically supplied and require the administration of local antibiotics for hours or days [7]. This solution achieves optimal concentrations for prevention and minimizes the adverse effects of systemic treatment. The use of surface coatings has been extensively investigated to prevent/treat orthopedic IAI, using either a non-degradable antibacterial surface or local release of antibiotics as solutions to coat the implant [3,8,9,10,11]. Local release of antibiotics from the biodegradable polymer coating requires sustained and controlled release of the antibiotic to inhibit microbial adhesion, colonization, and subsequent biofilm formation. In addition to its biodegradability and desired antibiotic release profile, the polymer system needs to have adequate mechanical strength and matrix formation [12].

Some of the approaches that have been widely used to prevent orthopedic IAI using local antibiotic release from coatings are calcium or silicon bone cements [13,14] and polymer hydrogels [7,8,15,16]. However, they have their limitations. Antibiotic-loaded cements have a burst release and limited release of embedded antibiotics (only 10% of the antibiotics, estimated) because of the diffusion through surface roughness, superficial pores, and surface erosion. However, the release of the antibiotic in the hydrogels is steady and is closely related to the crosslinking structure [3].

Hybrid sol-gel coatings are an example of this last-mentioned alternative. Sol-gel technology is a versatile method used to produce a wide diversity of materials. Among the advantages it offers are the simple sol-gel processing conditions and the possibility of tuning organic-inorganic hybrid materials for specific requirements [17,18]. Some studies have previously been conducted in which antibiotics are introduced into biodegradable sol-gel coatings, but these investigations are very scarce [19,20]. T. Nichol et al. [21] developed a sol-gel coating loaded with gentamicin for cementless hydroxyapatite-coated titanium orthopedic prostheses. They controlled the release of the antibiotic within the desirable time frame of 48 h. S. Radin et al. [19] synthesized sol-gel coatings loaded with vancomycin and studied the effect of the processing parameters on the coating degradation and antibiotic release.

In previous works, we have studied the effect of adding fluconazole and anidulafungin to a sol-gel coating in terms of electrochemical [22] and microbiological characterization [23]. Besides, in vitro studies and an in vivo model have been carried out on a moxifloxacin-loaded sol-gel coating [24]. However, the effect of adding antibiotics on the sol-gel network has not been addressed. The addition of large organic molecules to a sol-gel synthesis can act in detriment to the cross-linking of the network, resulting in poor adhesion of the coating to the substrate. For this reason, this work systematically studies the incorporation of eight antimicrobials of different natures into sol-gel coatings. This first step in knowing if it is possible to successfully incorporate these antibiotics into sol-gel coatings is important to develop a technology with personalized treatments based on each patient’s infection. 

We also investigated the simultaneous incorporation of two antibiotics into the coating to broaden the antibacterial spectrum against Gram-positive and Gram-negative bacteria. This objective addresses the prevention of both monomicrobial and polymicrobial infections; moreover, introducing antibiotics with different mechanisms of action can lead to a synergistic effect that increases their action against the bacteria to be prevented, reducing the possibility of antibiotic-resistance emergence [5,25,26].

For optimal design and synthesis of the coatings used for the desired application, this research focuses on studying the following two key factors: crosslinking and the surface of the coatings.

Achieving a controlled and constant antibiotic release rate is essential for the targeted applications. In these coatings, the release rate of the antibiotics is conditioned by the coating degradation [18]. Therefore, the design and study of the crosslinking of the formulations are very important to estimate and control the release rate of the antibiotics.

The surface of the coatings is also an important factor and must be studied. The hydrophobicity and roughness of these have an important effect on the initial bacterial attachment as well as the osseointegration capacity [27]. In most previous studies, it has been determined that surface roughness is directly related to the degree of bacterial adhesion. In addition, hydrophobicity influences each microbe differently depending on its nature. For instance, *S. aureus*, the most common bacteria in orthopedic IAI, prefers hydrophobic surfaces to adhere to, according to some research [3].

This research pursues the main objective of studying the influence of the addition of different antimicrobials (especially cefoxitin and linezolid) on the chemical and surface properties of the sol-gel coatings. Achieving the incorporation of these antimicrobials without compromising the sol-gel network will allow us to propose this technology as a versatile processing method to synthesize personalized coatings to prevent local joint prosthesis infections. 

## 2. Materials and Methods

### 2.1. Biofunctionalized Coatings Using Antibiotics: Materials and Sample Preparation

Organic–inorganic hybrid coatings were synthesized from methacryloxypropyltrimethoxy silane (MAPTMS, 98%, Acros Organics, Thermo Fisher Scientific, Waltham, MA, USA) and tetramethyl orthosilane (TMOS, 98%, Acros Organics). Sols were prepared from a mixture of MAPTMS and TMOS with a 1:2 molar ratio as described by El Hadad et al. [28]. To enhance cellular proliferation, as demonstrated in a previous work [29], tris(tri-methylsilyl) phosphite (92%, Sigma Aldrich, St. Louis, MI, USA) was added to the sol. The molar ratio of silanes to phosphorus precursor was fixed at 50. Ethanol was added as a solvent to avoid phase separation and water as a reagent to initiate the hydrolysis reaction. Ethanol and water were added in stoichiometric amounts. All species were mixed before addition of water. After the dropwise addition of the aqueous solution, suspensions were stirred for 24 h in a glove box. 

Coatings were made by adding different antimicrobials to the formulation. Eight antimicrobials (7 antibiotics and 1 antifungal) were used as biofunctionalizers in the coatings. Antibiotics: gentamicin sulfate salt (GEN, Sigma Aldrich), cefoxitin sodium salt (FOX, Sigma Aldrich), vancomycin hydrochloride hydrate (VAN, Sigma Aldrich), dicloxacillin sodium salt monohydrate (DCX, Sigma Aldrich), clindamycin hydrochloride (CLI, Sigma Aldrich), ampicillin (AMP, Sigma Aldrich), and linezolid (LNZ, 98%, Acros Organics). Antifungal: Amphotericin B (AMB, Sigma Aldrich). The used concentration was correlated with the maximum amount of water solubility of the antibiotic for most incorporated antibiotics. However, AMP, LNZ, and AMB antibiotics are insoluble or poorly soluble in water, so ethanol was used to dilute them, and the used concentration was the highest without producing supersaturation. The concentrations used in the formulations are summarized in Table 1. These antimicrobials were previously dissolved or suspended in water before adding them to the mixture (part of the ethanol volume was used to dissolve them or improve the antimicrobial agent solubility). All reagents were used as received from Sigma-Aldrich and Acros Organics.

Titanium sample pieces of 15 mm diameter × 25 mm thickness, prepared by a conventional powder metallurgy route, described in a previous work [30], were used as substrates (TiPM). The variation in surface roughness affects the coating distribution, so substrates were treated before the film deposition to achieve homogeneous surface conditions. Prior to the application of the sol-gel coating, the substrates were ground with successively finer SiC paper up to 1000 grit size, cleaned ultrasonically with acetone and alcohol, and dried.

A dipping device (KSV instrument-KSV DC) with a controlled withdrawal speed was used for the film deposition. Plates were immersed into the dissolution at a rate of 200 mm/s and were immediately removed at the same rate. 

Finally, samples were dried at 60 °C for 60 min inside an oven. An example of the visual appearance of the coated pieces is shown in Figure S1. During the thermal treatment, the condensation of the remaining OH groups was promoted, and evaporation of the solvents occurred, resulting in the final network (xerogel).

The surface morphology and composition of the as-prepared coatings were assessed by scanning electron microscopy (Teneo FEI, W filament, Lincoln, NE, USA). The lateral area of the samples was covered by a Cu layer to increase the conductivity. Images were taken at low vacuum and applying 2 kV and 0.2 nA. Energy dispersive spectrometry (EDS) measurements were performed using an X-ray microanalysis system along with an Octane Plus detector (EDAX, Pleasanton, CA, USA) of 30 mm^2^ area, which allowed for semi-quantitative analysis of the chemical composition by applying 5 kV and 0.2 nA. 

Thickness of films was measured using an ultrasonic thickness NEURTEK instrument (Eibar, Spain). 

### 2.2. Biofunctionalized Sol-Gel Coatings with FOX and LNZ: Materials and Sample Preparation

The following two antibiotics were chosen to perform a more detailed characterization: FOX and LNZ. The selection was based on the broadening of the antibacterial spectrum of the coatings for gram-positive and gram-negative bacteria and the pursuit of a synergistic effect with the administration of both antibiotics. Three coatings were prepared for each antibiotic with different antibiotic concentrations. A coating including the maximum concentrations of the two antibiotics was also prepared. A coating without antibiotics was synthesized for comparison. The concentrations of the prepared coatings are listed in Table 2.

### 2.3. Chemical Characterization

The evolution of the hydrolysis-condensation reaction was monitored by Fourier-transform infrared (FT-IR) spectroscopy. Each sample was prepared by adding a drop of the synthesis to a pressed KBr disc. Spectra were recorded with a Thermo Scientific NICOLET iS50 FT-IR System (Waltham, MA, USA) at room temperature in absorbance mode, covering the mid-infrared range from 500 to 4000 cm^−1^ and with 4 cm^−1^ resolution. For each sol, three measurements were carried out.

Sols were dried at room temperature for 7 days and ground into powder in an agate mortar before the characterization by ^29^Si-NMR and TGA [31].

Solid-state ^29^Si-NMR spectroscopy was used to determine the Si-O-Si crosslinking densification after curing treatment. The spectra were recorded in a Bruker AVANCE 400 spectrometer (Billerica, MA, USA) equipped with fast Fourier transform unit. The frequency used was 79.48 MHz (9.4 T). Samples were spun at 10 kHz around an axis inclined 54°44′ with respect to the external magnetic field. The used pulse length was 5 μs (90° pulse), the relaxation delay was 10s and 6000 accumulations were acquired. Spectra were referenced to TMS.

For the thermogravimetric analysis (TGA), around 30 mg of the xerogel was placed on alumina crucibles. Spectra were recorded from 30 to 900 °C at a rate of 10 °C/min in air atmosphere (PerkinElmer, STA 6000 instrument, Waltham, MA, USA). Duplicate measurements were made on each sample. 

### 2.4. Surface Characterization

The surface morphology, composition, and thickness of the produced coatings were evaluated using SEM, EDS, and ultrasonic transducer respectively as described in Section 2.1.

The wettability of the coatings was determined by measuring the static contact angle of Phosphate-Buffered Saline (PBS) (pH = 7.4) onto sol–gel surfaces using an automatic contact angle meter (DATAPHYSICS OCA 20 Goniometer, DataPhysics Instruments GmbH, Filderstadt, Germany). A sessile drop of 3 μL was deposited on the surfaces at room temperature. The water contact angle was determined by the half-angle method. The value given is the mean of 6 measurements.

For the calculation of thickness and contact angle values, a statistical analysis was performed. Mean and standard deviation values were calculated using the one-way ANOVA statistical technique using as error protection method the Tukey HSD method, which provided a confidence limit of 95%.

The topographical features of the coated samples were inspected by atomic force microscopy (AFM) for scan area sizes of 5 × 5 and 40 × 40 µm^2^ with 512 × 512 pixels resolution. The AFM instrument used was an XE-150 Park System operated under non-contact mode in ambient conditions at a scan rate of 0.3 Hz. The used silicon cantilever tip (N-type, µmasch, USA) had a nominal radius of 8 nm. XEI software version 4.3.0 (Park System Corp., Suwon si, Korea) was used for surface roughness analysis and Gwyddion software version 2.54 (gwyddion.net (accessed on 30 October 2021)) was used for image treatment. Statistical comparisons were made using the one-way ANOVA test, with *p* = 0.05 as the minimal level of significance and Tukey test was used to identify differences between groups.

## 3. Results

### 3.1. Synthesis and Characterization of Biofunctionalized Coatings

The obtained sol in most of the formulations was transparent, but the sols with GEN, VAN, and FOX showed some turbidity. Besides having an adequate viscosity that facilitates the uniform coverage of the substrate, sols did not evidence the separation of phases.

**Figure 1:**
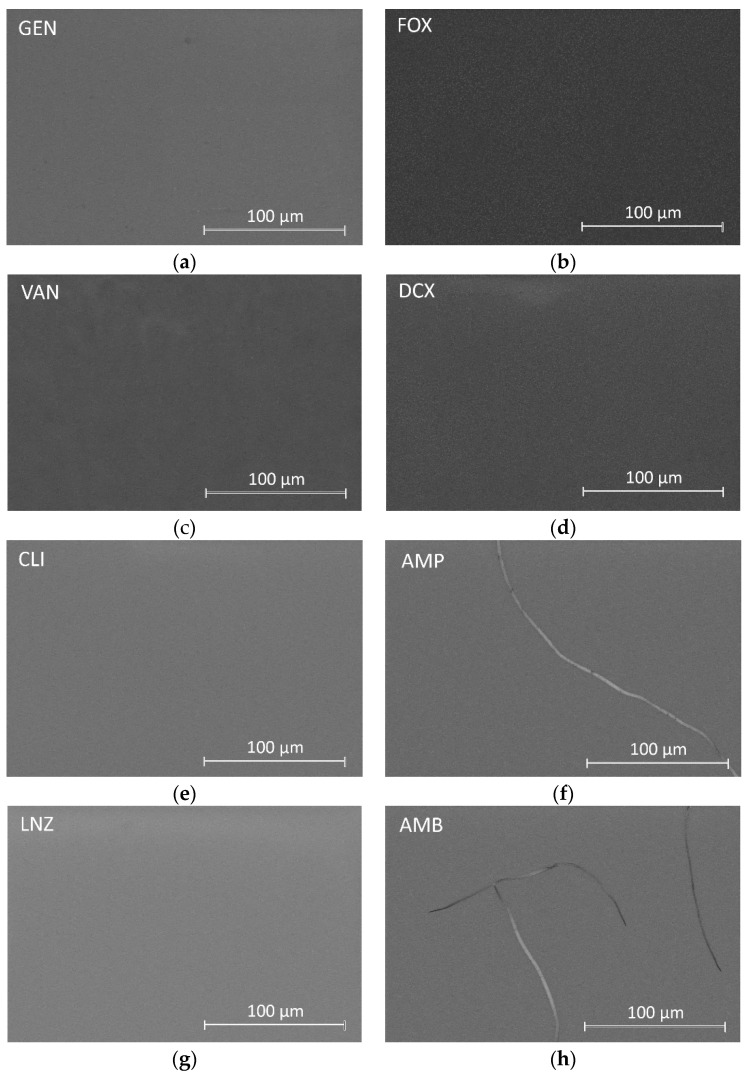
Micrographs obtained with CBS detector by SEM of the biofunctionalized coatings with different antibiotics: (**a**) gentamicin, (**b**) cefoxitin, (**c**) vancomycin, (**d**) dicloxacillin, (**e**) clindamycin, (**f**) ampicillin, (**g**) linezolid, and (**h**) amphotericin B at the concentrations described in Table 1.

Dried coatings were simple sight observed obtaining coatings without imperfections such as cracks or macropores. A more thorough inspection was performed using SEM. Figure 1 shows the micrographs of all formulations using the Backscatter Detector (Teneo FEI, Lincoln, NE, USA).

Inspection of the surfaces showed the formation of smooth, uniform, homogeneous, and crack-free coatings on the substrates in most formulations. In coatings containing AMP and AMB, isolated cracks were found throughout their surfaces. 

**Figure 2:**
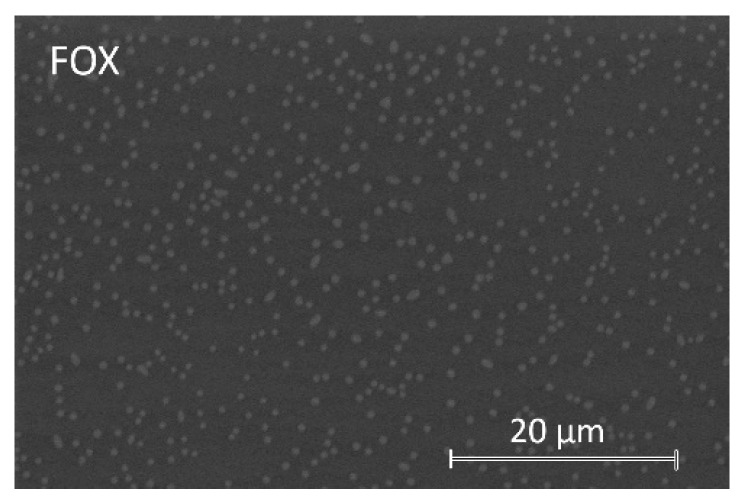
Micrograph of the biofunctionalized coating with the antibiotic cefoxitin (FOX) at a silanes:antibiotic molar ratio of 166:1 obtained with CBS detector by SEM.

On some of the surfaces of coatings, bright spots were observed distributed in a darker matrix, suggesting the presence of two well-differentiated phases. These coatings are those containing GEN, FOX, VAN, DCX, CLI, and LNZ. These bright spots change in size and quantity with each coating. The micrograph in Figure 2 is an example of such a surface observation on a FOX-containing coating.

When EDS analyses were performed in dark areas, the C, Si, and O elements were identified, corresponding to the organic precursors of the synthesis. EDS analyses on bright spots revealed the presence of P, corresponding to the organophosphite compound. In coatings containing FOX and DCX, Na was also identified in the bright spots, while in coatings with FOX, GEN, VAN, CLI, and LNZ, N was also detected in these spots.

**Figure 3:**
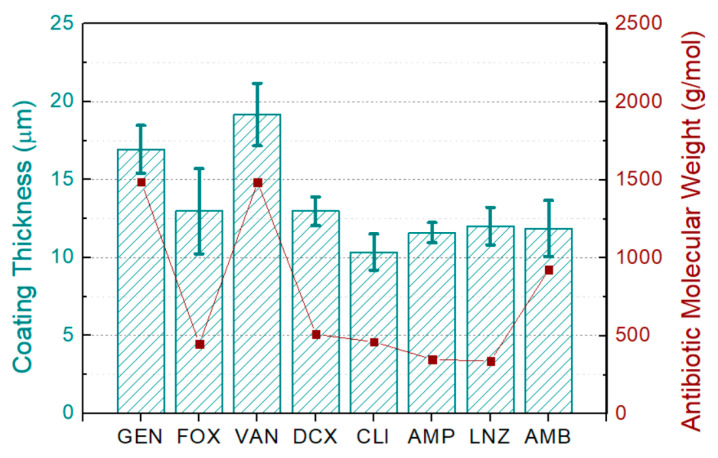
Correlation between the incorporated-antibiotics molecular weight in the coatings and their thicknesses.

Figure 3 shows the thickness of each coating and the molecular weight of the used antibiotics. Thicknesses ranged between 10 and 20 µm, with a median value of 12.5 µm. Significant differences between the obtained thicknesses were observed. 

### 3.2. Chemical Characterization of Coatings Loaded with FOX and LNZ

From this section, the characterization results carried out on the coatings loaded with FOX and LNZ at different concentrations are shown. 

**Figure 4:**
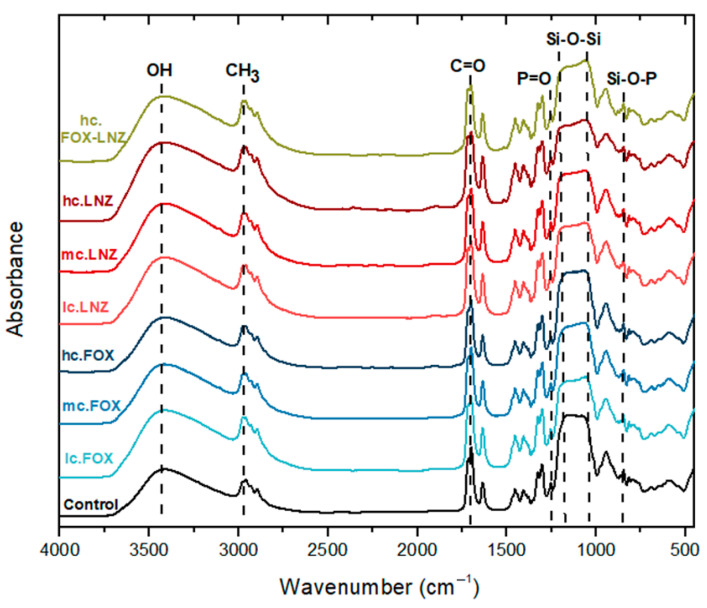
Representative FTIR absorption spectra of the studied sols containing FOX (hc., mc., and lc.), LNZ (hc., mc., and lc.), and FOX-LNZ (hc). As reference the spectrum of the control sol is depicted.

The sensitive analytical method of FTIR was used to study the polysiloxane network obtained in sols loaded with FOX and LNZ. Identification of the functional groups present in the formulations is possible with this technique. Furthermore, the detection of possible structural changes in the siloxane network due to the introduction of antibiotics or possible chemical bonds between antibiotics and the siloxane network could be observed. The FTIR spectra of the sols are plotted in Figure 4.

The formation of the silica network was evidenced by identifying the bands associated with the vibrational modes of the Si-O-Si chains, detected at ~815 cm^−1^ (weak band), ~1060 cm^−1^, and ~1160 cm^−1^; the Si-O-Si chains result from the condensation process. A broad band at ~3420 cm^−1^, related to the vibrational modes of OH groups, including those from SiOH formed through hydrolysis, was also observed. These bands proved the existence of hydrolysis and condensation phenomena [28,32,33].

The existence of the bands at 1254 and ~850 cm^−1^, related to the stretching vibration of a P=O bond [29] and the Si-O-P bending [34], respectively, was a clear indication of phosphorus presence and its incorporation into the silica network.

Absorption bands around 2960 and 2900 cm^−1^ were attributed to the presence of C–H bonds [32,35,36]. The bands at ~1720 cm^−1^ and ~1640 cm^−1^ were associated with the stretching vibrations of C=O carbonyl groups and the C=C groups of the methacrylate groups from the MAPTMS precursor, respectively [28]. The band at ~1450 cm^−1^ was attributed to the symmetrical and asymmetrical CH_3_ deformational modes [36]. The asymmetric and symmetric stretching vibrations of C–O and C–O–C bonds were attributed to bands at ~1320 and 1300 cm^−1^, respectively. Finally, the band at ~950 cm^−1^ was assigned to the C=C vibrations of the C=C–C=O group [28].

Neither differences in the shapes of the absorption bands nor the emergence of new bands were observed in the spectra.

The ^29^Si-NMR technique was used to study the siloxane network formed in each of the systems and their condensation degree. The chemical study was extended with this technique, as it offers more detailed information with better resolution thanks to the higher sensitivity to short-range interactions in comparison with the FTIR analysis. The ^29^Si-NMR analysis allowed the quantification of the crosslinking degree within the silicate network.

**Figure 5:**
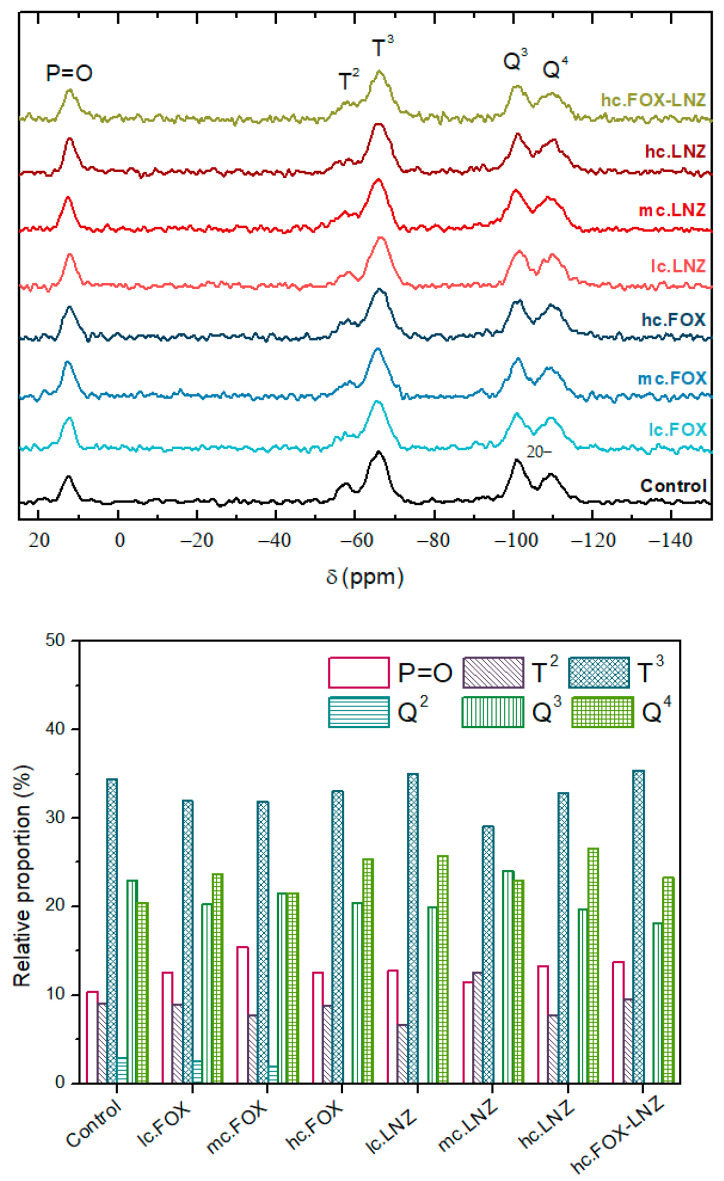
Solid-state ^29^Si-NMR spectra of each formulation (**up**). Relationship of the signals (**down**).

In ^29^Si-NMR spectroscopy, there is a well-established nomenclature for identifying each chemical shift of silicon. The T^n^ and Q^n^ structures represent the trialkoxysilane and tetraalkoxysilane functionality, respectively, while the superscript n indicates the number of produced siloxane bonds. In the case of the formulations presented in this study, T species were related to MAPTMS precursor and Q species to TMOS [28,36]. Table 3 lists all the possible signals that can be obtained in this case [29]. Figure 5 shows the solid-state ^29^Si-NMR spectra of the obtained xerogels.

The signals associated with T^2^, T^3^, Q^3^, and Q^4^ were observed in all xerogels at −57, −66, −101, and −110 ppm, respectively. In each of the systems, the achievement of a high degree of crosslinking of the siloxane network was observed. This statement was supported by the following observations:

- The absence of non-hydrolyzed species and species with a single siloxane bond in both precursors;
- The T^3^ predominance over the T^2^ signal in the MAPTMS precursor and the prevalence of the Q^3^ and Q^4^ signals in the TMOS precursor.

At ~12.5 ppm, another signal appeared, denoted as P=O in the spectra, that corresponds to the creation of a -P=O- bond in the entourage of the Si nuclei of the sol-gel network. The integration mechanism of this compound into the network was described elsewhere by Garcia-Casas et al. [29]. Briefly, the organophosphite undergoes a first reaction that leads to the oxidation of trivalent phosphorus to pentavalent phosphorus, allowing the hydrolyzation of the trimethylsilyl chain and its subsequent condensation.

In summary, these xerogels showed the formation of three-dimensional networks, dominated by T^3^ building blocks, accompanied by small amounts of P=O, T^2^, Q^3^, and Q^4^ units.

Table 4 summarizes the proportion of each detected signal in the systems. 

The antibiotics’ introduction slightly modified the contribution of each precursor to the crosslinking. An increase in the intensity of the P=O signal and attenuation of T^n^ and Q^n^ species were observed. Moreover, the T^n^ species intensity decreased more than the Q^n^ species signal. The number of fully condensed species increased in Q species. 

**Figure 6:**
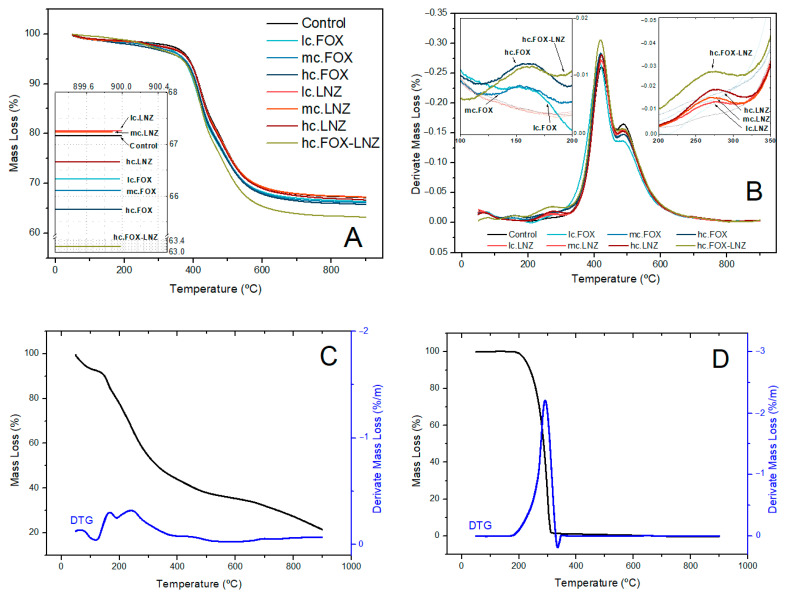
TGA (**A**) and DTG (**B**) profiles of the xerogels obtained during the syntheses. TGA and DTG profiles of the cefoxitin sodium Salt (FOX) (**C**) and linezolid (LNZ) (**D**).

Thermal characterization using TGA was performed to quantify the inorganic contribution of the network (Si-O-Si bonds). The thermogravimetric analysis (TGA) and the first derivative of the TGA (DTG) plots of the xerogels obtained during each synthesis are shown in Figure 6A,B. Thermogravimetric analyses of the antibiotics used are also shown in Figure 6C,D.

Thermogravimetric analysis of xerogels revealed a sharp inflection above 350 °C corresponding to the partial thermal degradation of organic matter (oligomers and unreacted organopolysiloxanes). This stage of degradation was accompanied by a subsequent stage (between 350 °C and 500 °C) due to the complete thermal degradation of the organic matter and water elimination from further silanol condensation [29]. Small losses after 800 °C in all samples are attributed to further burning of the residual organics [36].

A thermal degradation associated with the by-products elimination of the condensation (alcohol and water) and the unreacted reagents (pb_TMOS_ = 121 °C, pb_MAPTMS_ = 190 °C; pborganophosphite = 78–81 °C) occurred at temperatures below 350 °C [29]. The degradation of the antibiotics introduced into the formulations was another contribution present at these temperatures. Xerogels including FOX had a peak in DTG plots around 150 °C and those including LNZ around 275 °C, which coincided with the maximum thermal degradation of each antibiotic (Figure 6C,D).

The mass loss of the xerogels revealed that the hc.FOX-LNZ formulation decreased by almost 5 wt.% the inorganic contribution of Control (67.16% Control vs 63.21% hc.FOX-LNZ), see inset Figure 6A. The difference in mass loss between the rest of the formulations was only 1 wt.%.

### 3.3. Surface Characterization of Coatings Loaded with FOX and LNZ

**Figure 7:**
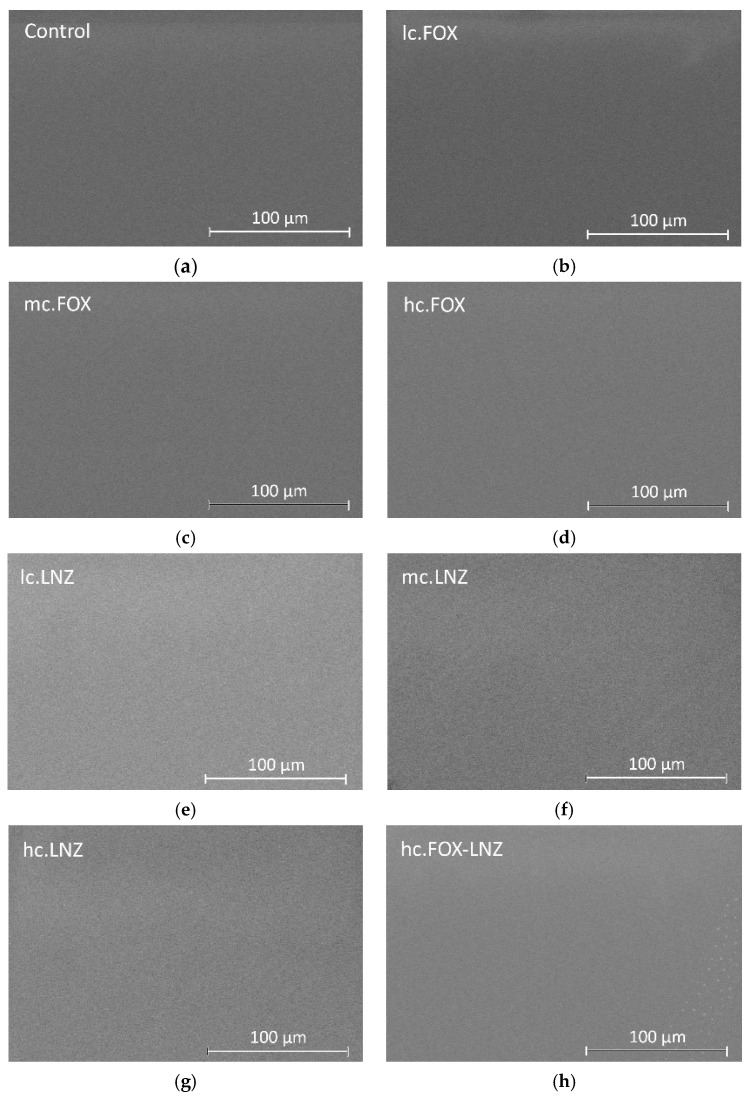
SEM micrographs for prepared coatings: (**a**) Control, (**b**) lc.FOX, (**c**) mc.FOX, (**d**) hc.FOX, (**e**) lc.LNZ, (**f**) mc.LNZ, (**g**) hc.LNZ, and (**h**) hc.FOX-LNZ deposited on TiPM substrates.

The surface of the synthetized coatings was inspected by SEM. Figure 7 shows SEM micrographs of the coatings using the Backscatter Detector. Inspection of the surfaces showed the formation of uniform, homogeneous, and crack-free coatings on the substrates.

**Figure 8:**
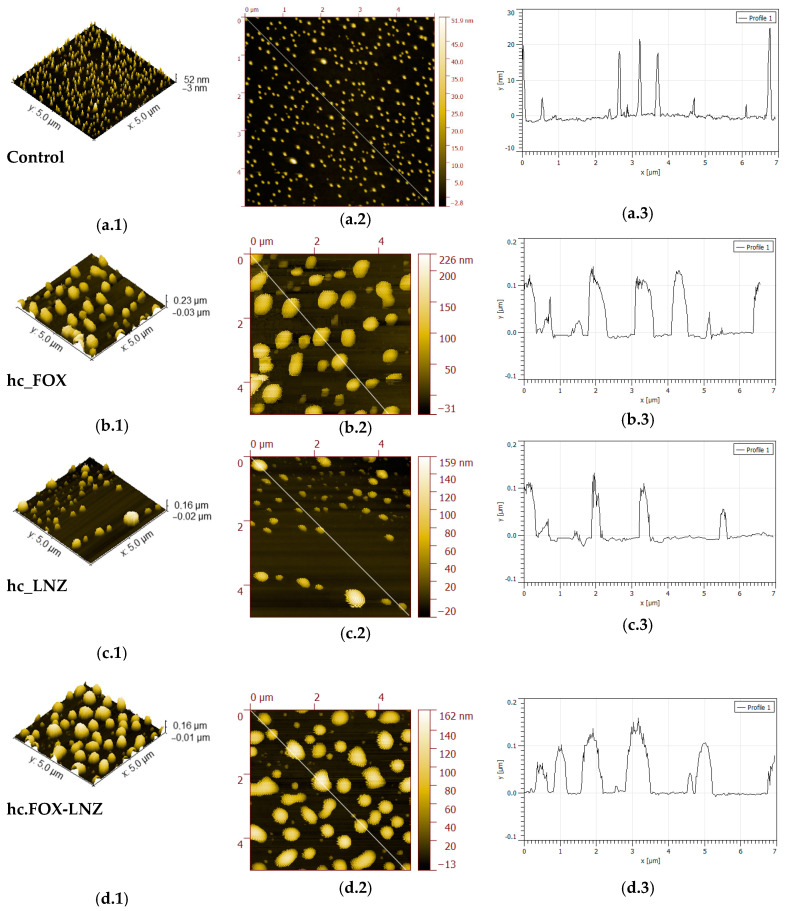
Representative non-contact mode AFM 3D topography images (**a.1**), (**b.1**), (**c.1**), and (**d.1**) of the thin films prepared onto TiPM substrates by sol-gel dip coating with (**a.2**), (**b.2**), (**c.2**), and (**d.2**) respective 2D surface morphology and (**a.3**), (**b.3**), (**c.3**), and (**d.3**) section profiles recorded along de white line indicated (scan area = 5 × 5 µm^2^). From top to bottom: Control, hc.FOX, hc.LNZ, and hc.FOX-LNZ film measurements (Dark colors indicate depressions, light colors protrusions).

For a better understanding and a more detailed morphology study of the achieved coatings, AFM was used. Figure 8 compares 5 × 5 µm^2^ AFM images of the surface morphologies of the sol–gel coated samples. In general, topographical images for the antibiotic-loaded coatings displayed a microscale roughness with irregular-shaped and randomly grown granular surfaces, with a significant number of protrusions (hills) exhibiting heights in the range of 0.03–0.16 µm. On the other hand, the size of clusters in the Control coating (Figure 8(a.1–a.3)) was much smaller, with measured heights in the range of 5–25 nm.

**Figure 9:**
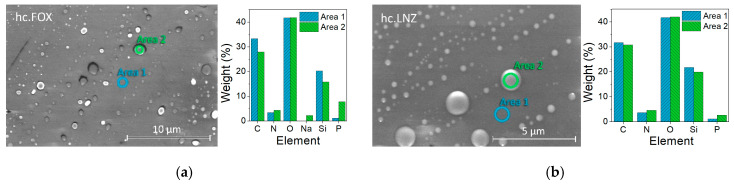
SEM/EDS micrographs for prepared coatings (**a**) hc.FOX (**b**) and hc.LNZ. (**Left**) SEM micrographs. (**Right**) Graphical results of EDS scan area on the indicated circles (green for the bright area and blue for the dark area).

A semiquantitative microanalysis was performed using SEM and EDS to confirm the protrusion’s identity. A very low vacuum with high magnifications was necessary to obtain these images (Figure 9). According to the obtained compositional analysis for both the hc.FOX and hc.LNZ samples, in the bright areas (corresponding to the protuberances) there was a decrease in the C and Si elements and an increase in N and P elements. In the case of hc.FOX, the Na element was present only in the bright areas.

In previous works where the Control coating was characterized, these formations were discovered and an increase in the size of these protuberances research was evidenced with the increase in the organophosphite concentration [29]. In this, in addition to the emergence of these protuberances associated with the organophosphite introduction, an increase in the concentration of elements associated with the antibiotics (Na or N) in these areas was also found. 

The surface texture of the coatings was studied by calculating several important roughness parameters from 40 × 40 µm^2^ scans. These parameters play an important role in the patient’s biological response to the prosthesis. Increasing implant-surface roughness is a critical factor for the implant fixation period and fixation strength with body tissues since it increases the values of cell adhesion, proliferation, and differentiation [36]. The numerical values for the most typical roughness parameters of the prepared coatings are summarized in Table 5. 

The lower roughness parameters (average roughness S_a_, RMS roughness S_q_, and maximum height S_z_) measured for the coatings proved that the coating application smoothed the surface compared to the bare TiPM substrate. This result supports SEM micrographs where homogeneity and non-existence of uncoated substrate areas in the coatings were apparent.

The values for these same roughness parameters were lower for the Control sample, confirming what was observed in the AFM images where the Control coating presents much smaller protrusions compared to the other coatings. 

The skewness (S_sk_) positive values expose that the surface peaks and asperities are predominant over valleys in the studied coatings. In addition, kurtosis (S_ku_) reports the sharpness of profile peaks that in all cases indicate the presence of inordinately high peaks.

Among the LNZ-containing coatings, no significant differences were found in any of the five roughness parameters studied. 

Among the coatings containing cefoxitin, lc.FOX was statistically different from mc.FOX and hc.FOX when comparing S_a_ and S_q_. For maximum height (S_z_), mc.FOX was different from hc.FOX.

**Figure 10:**
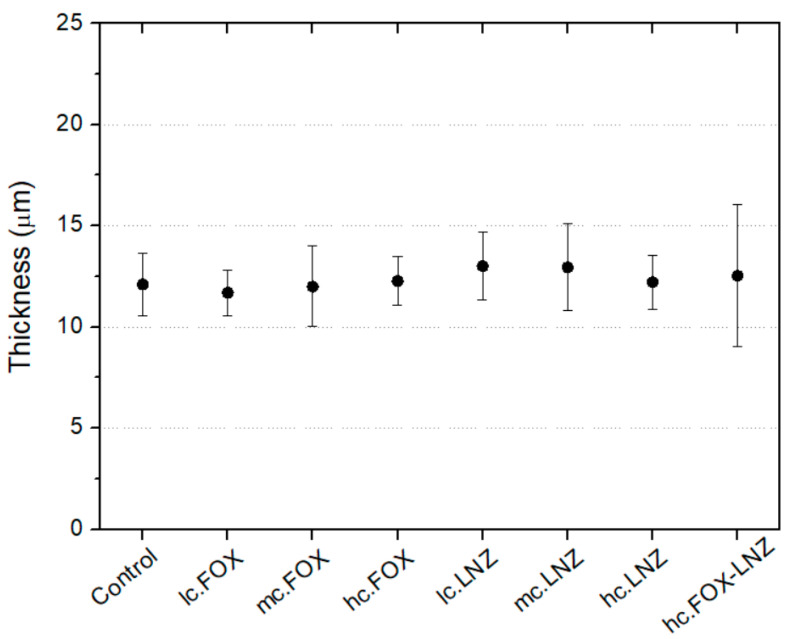
Thickness of sol-gel coatings prepared onto TiPM by dip-coating. No statistically significant differences were found between coatings (2-way ANOVA, *p* < 0.05). Bars indicate the standard deviations.

Figure 10 shows the coating thickness measurements. All the synthesized coatings had values between 10 and 15 μm without statistically significant differences between them.

**Figure 11:**
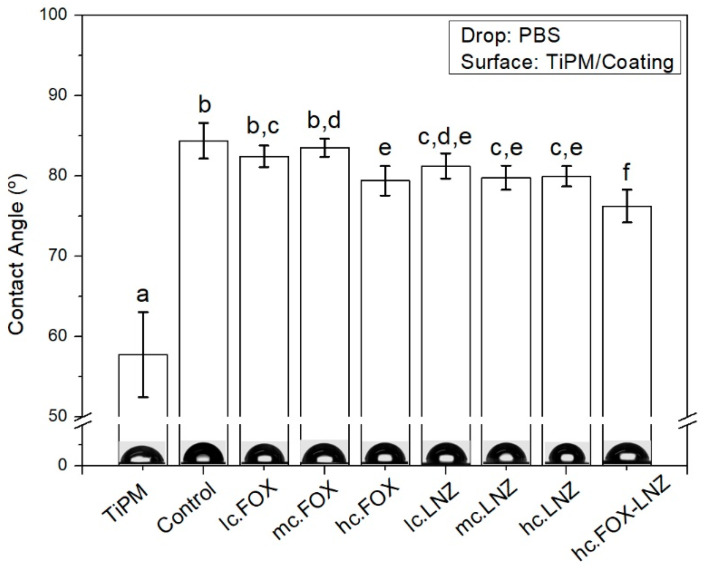
Contact angle results for films deposited on TiPM substrates. Bars with different letters denote statistical significance, *p* < 0.05 one-way ANOVA using Tukey HSD method. N = 10–12. All values are means ± SE.

The wettability of the formulations and the substrate were studied by means of contact angle measurements. The results are shown in Figure 11. The contact angle value was higher in all the coatings compared to the substrate as a result, among other factors, of the decrease in surface roughness. Furthermore, between the coatings, there were also significant differences. The most hydrophilic coating was the one with the combination of antibiotics. 

## 4. Discussion

In this study, eight antibiotics (GEN, FOX, VAN, DCX, CLI, AMP, LNZ, AMB) were separately introduced into organic-inorganic sol-gel coatings for TiPM metal prostheses. Furthermore, coatings with different FOX and LNZ concentrations (separately and mixed) were synthesized and examined in terms of morphology and chemical characteristics.

The formation of smooth, uniform, homogeneous, and crack-free coatings was successfully achieved after introducing most of the antibiotics. Isolated cracks were found in coatings loaded with AMP and AMB, possibly related to the poor solubility or insolubility of these antibiotics in water.

The obtained differences in the thickness of the coatings could be related to multiple factors such as molecular weight, chemical nature, solubility, and concentration of the incorporated antibiotic. Although more testing would be needed to determine the contribution of each of these factors to the final thickness, the molecular weight is a highly influential factor; this can be observed in the five coatings (GEN, FOX, VAN, DCX, CLI) with a very similar amount by weight of added antibiotic in the sol-gel and yet markedly different thicknesses, which correlate well with the molecular weight, increasing for higher molecular weight antibiotics (GEN and VAN), as shown in Figure 3.

Coatings had a microscale roughness with irregular-shaped and randomly grown granular surfaces. These granular growths, together with the matrix, corresponded to two well-differentiated micro-phases. The EDS result indicates that the hills (bright areas) were the result of antibiotic and organophosphite accumulations in these areas. NMR analysis supports this claim since the increase in the number of P=O bonds in the network crosslinking occurred in formulations containing antibiotics. With these results, it could be theorized that cluster growth is due to a spontaneous organization in domains or micro-phases (“two-phase systems”), obtaining inorganic segments of bioactive silica-rich regions (matrix) and organic segments of methacryloxypropyl chain and P-O-Si bonds-rich regions (protrusions). The antibiotics would be housed within the organic segment, manifesting in a greater volume in that area. This organization could be possible due to non-covalent interactions (e.g., hydrogen bonds, Van der Waals forces, electrostatic forces, interactions) without external intervention. The factors that could influence this organization are the molecular weight of the introduced antibiotic and, possibly, its hydrophilicity. In the antibiotic-free coating, these microphases were evidenced, with the organophosphite being the contributor to the granular formations, although significant differences were found between the size of the protrusions in this coating and the antibiotic-laden coatings. This result shows that the antibiotics only lodge in these protrusions, being able to favor their formation and increase their size, but the spontaneous organization is not due to their introduction.

In fact, chemical studies suggested the absence of chemical bonds between antibiotics and the siloxane network, despite the slight modification of the network crosslinking caused by the antibiotic introduction. FT-IR results inferred the apparent non-modification of the siloxane network due to the introduction of antibiotics and the absence of chemical bonds between antibiotics and the siloxane network. However, bands related to the introduced antibiotics may be present, but the low concentrations of the antibiotics would only result in very weak bands, if any, being indistinguishable from the main bands of the silicate network.

Solid-state ^29^Si-NMR results suggested the favoring of the participation of the phosphorus-based compound in the network crosslinking when an antibiotic is introduced. This phenomenon could be explained because the antibiotics represent a steric hindrance in the network and both, tris(tri-methylsilyl) phosphite and MAPTMS (due to its long organic chain), give the antibiotic room to accommodate. 

TGA results, as FT-IR and NMR results, indicated a decrease in network crosslinking in formulations with antimicrobials. Despite all the formulations presenting a greater mass loss compared to Control sample, in most of the cases, it is difficult to elucidate any conclusion on this matter since it was only a 1 wt.% difference. The apparent differences in mass loss could be related to the antibiotic-loaded amounts and their influence on the network crosslinking. The peaks coinciding with the degradation of the antibiotics evidenced that with increasing their concentration, a greater contribution to the degradation occurred (see insets Figure 6B).

Despite these chemical studies, being able to demonstrate the influence or chemical bonds between the antibiotics introduced and the siloxane network is not a straightforward task. The large difference between the number of moles of both parts during the synthesis is the main obstacle.

The relationship between concentration, the introduction of antibiotics, and the size or distribution of protrusions was unclear. From the AFM analysis, no behavior was found that relates to all these parameters. These protuberances behaved quite randomly in coatings loaded with antibiotics. However, all the resulting surface morphologies in the antibiotic coatings were very promising in terms of decreasing microbial adherence. Previous studies proved that these morphologies have an antimicrobial effect only related to their microstructure [37,38].

The wettability differences found between coatings could be attributed to their roughness. In contact angle measurements where the deposited drop has a low viscosity, it is reported that the increase in roughness decreases the contact angle because of more surface area [32]. These results can be contrasted with the obtained roughness parameters, observing an opposite trend between both parameters. Wettability is a key factor affecting not only protein adsorption and cell attachment but also the degradation kinetics of coatings when in contact with a physiological medium. Hydrophobic surfaces or surfaces with an intermediate wettability (60–90°) are the suggestion by most literature to achieve good biocompatibility through protein adsorption and to prevent bacterial adhesion [39,40,41].

In coatings loaded with FOX and LNZ (separately), no conclusions could be reached about the relationship between concentration, type of introduced antibiotics, and size or distribution of protrusions. Neither were there great differences found in network crosslinking, roughness, thickness, or wettability. However, the coating loaded with the two antibiotics simultaneously exhibited weaker network crosslinking along with a more hydrophilic surface than the rest. This result indicates that the concentration of antibiotics can influence the final characteristics of the coating, although it is necessary to vary the concentration considerably to obtain significant differences.

The roughness and hydrophilicity results make the coating loaded with the two antibiotics a good candidate for combined therapy to fight different bacteria, including *S. aureus*. However, in the next step, biological assessments will be needed to shed more light on the antibacterial effectiveness of the coating.

## 5. Conclusions

The introduction of different antibiotics into biodegradable sol-gel coatings for metal joint prostheses was successfully carried out. Sol-gel formulations were designed as carriers for different antibiotics separately (GEN, FOX, VAN, DCX, CLI, AMP, LNZ, AMB) and for two antibiotics simultaneously (FOX and LNZ). Synthesis containing either AMP or AMB must be optimized to obtain crack-free coatings.

The surface of coatings contains a “two-phase system” with an inorganic silica-rich matrix and organic-rich protrusions. The granular surfaces shown in the coatings resulted in microscale roughness and were attributed to the accumulation of antibiotics and organophosphites in the surface protrusions. The existence of chemical bonds between the introduced antibiotics and the siloxane network could not be demonstrated, although the presence of the antibiotics indirectly modified the network crosslinking. The influence of antibiotic introduction on network crosslinking, roughness, thickness, and wettability is evidenced only for significant variations of introduced concentrations.

The coating loaded with two antibiotics showed somehow a weaker crosslinking. However, introducing two antibiotics broadens the antibacterial spectrum of the coating, resulting in a good strategy to prevent local joint prosthesis infections. Thus, further studies are required to verify the antibacterial activity of those coatings.

## Figures and Tables

**Table 1:** Molar ratio between the concentration of the silanes and the antibiotics used in the sol-gel formulations.

Antibiotic	Molar Ratio Silanes:Antibiotic
GEN	540:1
FOX	166:1
VAN	550:1
DCX	185:1
CLI	172:1
AMP	682:1
LNZ	165:1
AMB	902:1

**Table 2:** List of the identifying names of the coatings according to the doped antibiotic and its concentration.

Denotation ^1^	Antibiotic Concentration (mmol)
Control	Non-Antibiotic
lc.FOX	0.037 mmol FOX
mc.FOX	0.073 mmol FOX
hc.FOX	0.147 mmol FOX
lc.LNZ	0.037 mmol LNZ
mc.LNZ	0.074 mmol LNZ
hc.LNZ	0.148 mmol LNZ
hc.FOX-LNZ	0.147 mmol FOX + 0.148 mmol LNZ

^1^ lc: lowest concentration, mc: medium concentration, hc: highest concentration.

**Table 3:** Chemical shift (δ) of TMOS and MAPTMS employing silicon nuclei.

Signal	Nature of Silicon Unit	Precursor	δ (ppm)
T^0^	Unhydrolyzed	MAPTMS	−42
T^1^	Once condensed (one siloxane bond)		−49
T^2^	Doubly condensed (two siloxane bonds)		−58
T^3^	Fully condensed (three siloxane bonds)		−67
Q^0^	Unhydrolyzed	TMOS	−82
Q^1^	Once condensed (one siloxane bond)		−86
Q^2^	Doubly condensed (two siloxane bonds)		−92
Q^3^	Triple condensed (three siloxane bonds)		−101
Q^4^	Fully condensed (four siloxane bonds)		−110

**Table 4:** Relative proportions of T and Q species in the organic-inorganic hybrid materials from the solid-state ^29^Si NMR spectra in Figure 5.

Sample	Proportions ^a^ (%)						Relative ^b^ Proportions (%)		Relative ^c^ Proportions (%)			Ratio ^d^ (%)		
	P=O	T^2^	T^3^	Q^2^	Q^3^	Q^4^	T^2^	T^3^	Q^2^	Q^3^	Q^4^	P=O	T^n^	Q^n^
Control	10.37	9.00	34.44	2.89	22.89	20.41	20.72	79.28	6.26	49.56	44.18	10.37	43.43	46.20
lc.FOX	12.55	9.00	31.92	2.58	20.25	23.70	21.99	78.01	5.54	43.53	50.93	12.55	40.91	46.54
mc.FOX	15.45	7.73	31.88	1.90	21.51	21.53	19.53	80.47	4.22	47.86	47.92	15.45	39.61	44.94
hc.FOX	12.50	8.78	32.99	-	20.42	25.31	21.03	78.97	-	44.64	55.36	12.50	41.77	45.73
lc.LNZ	12.75	6.64	35.04	-	19.91	25.66	15.94	84.06	-	43.69	56.31	12.74	41.69	45.57
mc.LNZ	11.51	12.55	29.02	-	23.98	22.94	30.19	69.81	-	51.12	48.88	11.51	41.57	46.92
hc.LNZ	13.26	7.73	32.78	-	19.67	26.56	19.09	80.91	-	42.55	57.45	13.26	40.51	46.23
hc.FOX-LNZ	13.78	9.56	35.36	-	18.06	23.24	21.28	78.72	-	43.73	56.27	13.78	44.92	41.30

^a^ Peak area % was calculated by the deconvolution technique. Error value assumed is ±5%. ^b^ (Each T species/total T species) × 100%. ^c^ (Each Q species/total Q species) × 100%. ^d^ Si-P = (Si-P signal/total signals) × 100%, T^n^ = (total T species/total signals) × 100%, Q^n^ = (total Q species/total signals) × 100%.

**Table 5:** Surface roughness parameters and their standard deviations obtained by AFM images analysis.

Surface	S_a_ (nm)	S_q_ (nm)	S_z_ (µm)	S_sk_ (µm)	S_ku_ (µm)
TiPM	169.79 ± 42.80	227.96 ± 45.05	2.111 ± 0.564	0.659 ± 1.548	6.330 ± 3.872
Control	9.61 ± 2.43	12.43 ± 3.03	0.087 ± 0.025	0.493 ± 0.674	3.809 ± 1.248
lc.FOX	22.30 ± 5.45	29.21 ± 5.85	0.322 ± 0.200	1.865 ± 2.323	17.479 ± 26.406
mc.FOX	49.58 ± 8.22	76.29 ± 9.42	0.642 ± 0.116	2.393 ± 0.915	10.741 ± 3.080
hc.FOX	51.83 ± 13.53	79.30 ± 27.82	1.014 ± 0.690	2.707 ± 2.541	23.482 ± 27.116
lc.LNZ	63.60 ± 27.16	131.92 ± 72.30	1.630 ± 0.760	4.864 ± 2.014	36.828 ± 24.462
mc.LNZ	43.85 ± 15.36	89.18 ± 40.28	1.488 ± 0.620	5.580 ± 2.802	58.149 ± 32.564
hc.LNZ	38.95 ± 13.84	67.85 ± 55.44	0.870 ± 0.748	2.553 ± 3.185	26.995 ± 34.596
hc.FOX-LNZ	62.04 ± 21.79	127.09 ± 92.40	1.467 ± 0.959	4.429 ± 3.075	39.812 ± 34.642

S_a_ = arithmetic mean height; S_q_ = root mean square height; S_z_ = maximum height; S_sk_ = skewness and S_ku_ = kurtosis of the surface. The values are average of 8 measurements (maps of 40 × 40 µm).

## Data Availability

Not applicable.

## Supplementary Materials

The following supporting information can be downloaded at: https://www.mdpi.com/article/10.3390/ma15144752/s1, Figure S1: Visual appearance of the powder metallurgical titanium pieces coated with the sol-gel formulations.
