# Supplementary figures and images for: Influence of Addition of Antibiotics on Chemical and Surface Properties of Sol-Gel Coatings
Source: Materials (Basel). 2022 Jul 7;15(14):4752. doi: 10.3390/ma15144752 (PMC9317242; doi:10.3390/ma15144752)

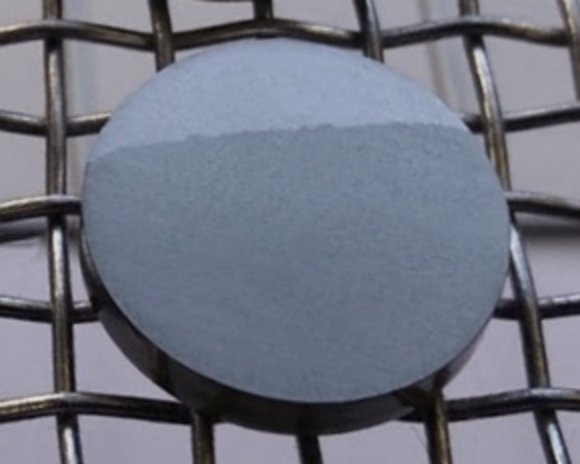

Supplement: Supplementary file 1 [file materials-15-04752-s001.zip › materials-1785458-supplementary.pdf]
